# Epidemiologic case investigation on the zoonotic transmission of Methicillin-resistant *Staphylococcus pseudintermedius* among dogs and their owners

**DOI:** 10.1016/j.jiph.2023.10.041

**Published:** 2023-12

**Authors:** Luciana Guimarães, Izabel Mello Teixeira, Isabella Thomaz da Silva, Milena Antunes, Camilla Pesset, Carolina Fonseca, Ana Luiza Santos, Marina Farrel Côrtes, Bruno Penna

**Affiliations:** a Laboratório de Cocos Gram positivos, Instituto Biomédico, UFF, Brazil; b Graduate Program in Microbiology, Instituto de Microbiologia Paulo de Goes, UFRJ, Brazil; c Laboratório de Investigação Médica LIM49, Instituto de Medicina Tropical, USP, Brazil; d Graduate Program in Veterinary Medicine, Fluminense Federal University, Brazil

**Keywords:** *Staphylococcus pseudintermedius*, MRSP, Zoonotic transmission

## Abstract

Dogs often carry methicillin-resistant Staphylococci asymptomatically. These bacteria are frequently linked to conditions such as canine pyoderma and otitis. Close interaction between dogs and humans can facilitate the exchange of resistant strains, particularly Methicillin-resistant *Staphylococcus pseudintermedius* (MRSP). This represents a public health issue, since these strains, in addition to occasionally causing infections in humans, can also serve as a source of resistance and virulence genes for strains of greater importance in human medicine, such as *Staphylococcus aureus*. Furthermore, MRSP strains are often multidrug resistant, which ends up compromising the treatment of infections. This study aimed to assess the potential transmission of *Staphylococcus pseudintermedius* among dogs and their owners. We examined a total of one hundred canine samples collected from cases of pyoderma and otitis to detect the presence of staphylococci. Simultaneously, we conducted evaluations on all dog owners. Staphylococci strains were identified using MALDI-TOF MS and PCR targeting the *nuc* gene. Methicillin resistance screening was also performed by detecting the *mecA* gene using PCR. Among the sampled dogs, 64 carried *S. pseudintermedius*. Nine were identified as MRSP. In six instances, dogs and their owners exhibited *S. pseudintermedius*. These samples underwent genome sequencing and were screened for antimicrobial resistance genes, *SCCmec* typing, MLST characterization, and Single Nucleotide Polymorphisms (SNP) analyses. The results of the phylogenetic analysis revealed that in three cases, dogs and owners had closely related isolates, suggesting interspecies transmission. Two of these cases involved MRSP and one MSSP. Moreover, in the two MRSP cases, the same SCC*mec* type (type V) was detected. Additionally, the sequence type was consistent across all three cases involving dogs and owners (MSSP ST2277, MRSP ST2282, and ST2286). These findings strongly indicate a transmission event. Since *Staphylococcus pseudintermedius* is primarily isolated from canine samples, it is plausible that dogs may have acted as a potential source. In the remaining three cases, despite identifying the same species in both samples, they had notable phylogenetic differences.

## Introduction

*Staphylococcus pseudintermedius* is an integral component of the inherent skin microbiota in dogs [1]. This bacterium assumes the role of an opportunistic pathogen, accountable for various infections within companion animals like pyoderma, otitis, and infections affecting wounds and the urinary tract [2]—Notably, the range of diseases attributed to *S. pseudintermedius* parallels that caused by *S. aureus*.

The issue of methicillin resistance has evolved into a substantial concern within staphylococci, bearing noteworthy implications for both animals and public health [3]. The prevalence of methicillin resistance in *Staphylococcus pseudintermedius* (MRSP) has exhibited a global surge [4], [5], tracing back to its initial documentation in North America [6]. The unwarranted employment of antimicrobials in companion animals is a driving force behind the escalation of this resistance [7].

Infections stemming from MRSP pose intricate therapeutic challenges. These isolates commonly demonstrate resistance to additional antimicrobial classes, including aminoglycosides, macrolides, and fluoroquinolones, thereby amplifying the complexities of treatment [8], [9]. Previous investigations have unveiled a substantial genetic diversity among *S. pseudintermedius*, with over 1400 sequence types (STs) documented, with consensus prevailing around the prominence of ST71, ST68, and ST45 as the most prolific MRSP clones [9]. The lineage of ST71, initially identified in Europe, has achieved widespread dissemination [10]. Notably, recent findings have pinpointed the diffusion of ST71 in the region of Rio de Janeiro [11].

Furthermore, several studies have documented zoonotic transmission of *S. pseudintermedius* and even MRSP from dogs to humans [12], [13], [14]. The proximity of contact between dogs and humans serves as a facilitating factor for such transmissions [15]. Notably, *S. pseudintermedius* can successfully colonize human skin by suppressing the growth of indigenous bacterial microbiota. This represents a public health problem, since these strains, in addition to occasionally causing infections in humans, can also serve as a source of resistance and virulence genes for strains of greater importance in human medicine, such as *S. aureus*. Furthermore, MRSP strains are often multidrug resistant, which ends up compromising the treatment of infections [7], [13].

Additionally, the potential for misidentification looms over *S. pseudintermedius* human infections, as they might erroneously be classified as *Staphylococcus aureus* [16], [17] or *Staphylococcus intermedius* [18] due to shared characteristics. The present study delved into the investigation of cross-contamination scenarios involving methicillin-resistant and methicillin-susceptible strains of *Staphylococcus pseudintermedius* among dogs and their respective human owners.

## Material and methods

### Bacterial isolates and identification

212 male or female samples were obtained from 100 adult dogs (ages 1–17 years). Forty-seven samples were obtained from 47 dogs affected by pyoderma (papules, pustules, circular crusts, dry or flaky patches of skin). One hundred sixty-five samples were collected from 53 dogs with pyoderma and otitis externa (skin redness, swelling, scratching, increased discharge, and scaly skin). All dogs were attended in a veterinary dermatology service from different veterinary clinics in Rio de Janeiro, Brazil. All samples were processed to detect *Staphylococcus pseudintermedius*. A total of 100 samples were collected from owner’s nasal fossa. All samples were collected using sterile cotton swabs (Copan Diagnostic, Italy) from January to December 2018. In animals affected by pyoderma, samples were collected, mainly from unruptured pustules and simultaneously from the ears of animals with otitis (unilateral or both). In the owners, the samples were collected by themselves in the nasal cavity. All samples were collected with authorization and approval from their respective ethics committees (CEUA-UFF:3023311020/ CAAE-UFF:14348319.4.0000.5243).

At the time of collection, a form for animals was also filled out, including relevant data for the study such as age, gender, previous use of antimicrobials, and sleeping location. For the owners, information regarding age, gender, presence of immunosuppressive diseases, previous use of antimicrobials, and workplace location. Swabs were seeded into Salt Mannitol Agar (KASVI, Italy) and incubated aerobically at 37 °C for 24 h. All the isolates were first identified with Matrix-assisted laser desorption ionization-time of flight mass spectrometry (MALDI-TOF MS) using the Microflex system (Bruker Daltonics, Bremen, Germany) according to the manufacturer's instructions. To confirm the species of the group SIG (*Staphylococcus intermedius* group), PCR was performed to detect the *nuc* gene of the samples from the methodology of Sazaki et al., 2010 [19]. PCR was performed using Promega Kit (GoTaq® G2 DNA Polymerase), and reagents concentrations were 5X Green Reaction Buffer, 0,2 mM of dNTPs, 1.5 mM of MgCl_2_, ten pmol of each primer (tabela:1), 2 U of GoTaq, and 100 ng of DNA template in a total reaction volume of 25 µl. A sample of *S. pseudintermedius* (ED99) was used as a positive control for all reactions.

### DNA extraction

DNA of all *Staphylococcus pseudintermedius* samples were obtained using Chelex® (Bio-Rad, Califórnia, USA) and according to the methodology described by Walsh et al., 1991 [20], with modifications. In a microtube containing 99 µl of Chelex (5%) and 1 µl of Proteinase K (2%) (Ludwig, Rio Grande do Sul, Brasil) were added 4–5 bacterial colonies. The microtube was then vortexed and incubated at 56 ºC for 1 h in a dry bath. After this incubation period, the microtubes were again shaken (vórtex) and set at 100 ºC for 10 min in a dry bath. Then, in this new incubation, the microtubes were centrifuged at 16.000 xg for 2 min. We then transferred the supernatant to another correctly identified microtube and reserved it at − 20 ºC for later use in amplification reactions.

### Detection of the *mecA* gene

To detect the *mecA* gene, PCR was conducted according to the recommendations of Zhang et al., 2005 [21], using the same Promega PCR kit. Primers used in all the reactions are listed in Table 1. PCR was performed in a Veriti thermocycler (Applied Biosystems, California, USA). We visualized products under ultraviolet light after the products were stained with Gel Red (Invitrogen). We used a 1Kb molecular size marker (Invitrogen).

**Table 1:** SNPs analysis for the pairs of samples of *S. pseudintermedius* grouped in the phylogenetic tree isolated from dogs in Rio de Janeiro.

Sample	Host	Isolation site	SNP
19SD1	Dog 19	dermal secretion	59
19T1	Owner 19	nasal swab	
82SDR1	Dog 82	dermal secretion	86
82TR1	Owner 82	nasal swab	
85SAD2	Dog 85	ear secretion	93
85T3	Owner 85	nasal swab	
55SAER1	Dog 55	ear secretion	152
55SAER3	Dog 55	ear secretion	
22SAE2	Dog 22	ear secretion	205
22SDR2	Dog 22	dermal secretion	
56SADR2	Dog 56	ear secretion	304
57SDR1	Dog 57	dermal secretion	
1SD1	Dog 1	dermal secretion	7961
18SD1	Dog 18	dermal secretion	
17SD1	Dog 17	dermal secretion	9220
27T21	Owner 27	nasal swab	

### Whole-genome sequencing and Genome Assembly

For whole-genome sequencing, we analyzed only one strain from each dog and the respective pair of human origin, previously identified as *S. pseudintermedius*. Samples were cultured in Tryptone Soy Agar (KASVI, Italy) and incubated aerobically at 37 ºC for 24 h. According to the manufacturer’s instructions, DNA extraction was conducted using the Wizard Genomic DNA Kit (Promega, Madison, USA). The extracted DNA was quantified using Quantus Fluorometer (Promega, Madison, EUA) according to the manufacturer's instructions.

Library preparation was performed using Illumina DNA prep (M) Tagmentation (96 samples) and Nextera DNA CD index (96 indexes, 96 samples) (Illumina, San Diego, California, USA), according to the manufacturer’s instructions. Libraries were subsequently quantified using Qubit and dsDNA HS-kit (Thermo Fisher, Waltham, Massachusetts, USA). Finally, the sample was loaded on a HiSeq2500 system and ran for 201 cycles (PE125), pair-end (500 bp library) using HiSeq Rapid SBS Kit v2 chemistry. The reads obtained were trimmed using BBDuk with default parameters. We verified the quality of the sequence files with fastQC. Reads were trimmed using trimmomatic v0.38. Then, fastq files were assembled de novo using SPADES. Additionally, after a BLAST search, the closest genome in the genebank was used as a reference (HSP125, accession number NZ_CP066708.1) for a new assembly using BWA MEM, bowtie2, and UGENE v. 45.1 [22].

A phylogenetic tree was constructed using the REALPHY tool (v. 1.12) using 55 genomes based on Single Nucleotide Polymorphisms (SNP) with default parameters [23]. Briefly, the sequences were aligned via bowtie2. From these alignments, phylogenies were reconstructed using the maximum likelihood method PhyML with a GTR substitution matrix and a gamma-distributed rate heterogeneity model. The tree was visualized and edited using iTOL V 6.6 [24]. SNPs were calculated by realigning each genome pair using bowtie2 and analyzing with Seaview [25]. A synteny analysis was performed for the pairs using ProgressiveMauve version 2.4.0 [26], and the circular genome alignment was generated using BRIG [Alikhan NF, Petty NK, Ben Zakour NL, Beatson SA. BLAST Ring Image Generator (BRIG): simple prokaryote genome comparisons. BMC Genomics. 2011 Aug 8;12:402. Doi: 10.1186/1471–2164–12–402. PMID: 21824423; PMCID: PMC3163573.]. MLST typing was performed in silico using the MLST tool from the Center for Genomic Epidemiology (CGE) (https://cge.food.dtu.dk/services/MLST/). SCC*mec* elements were identified using the CGE tool SCC*mec*Finder (https://cge.food.dtu.dk/services/SCCmecFinder/). The genetic determinants of resistance were searched using two tools: ResFinder from CGE (https://cge.food.dtu.dk/services/ResFinder/) and The Comprehensive Antibiotic Resistance Database – CARD (https://card.mcmaster.ca/home).

### Statistical analysis

Statistical analysis was conducted using GraphPad software (8.0.1), and the methodology used was Fisher's Exact Test. Values of P < 0005 were considered statistically significant.

## Results

We identified *Staphylococcus pseudintermedius* from sixty-four dogs (64%). Among these animals, nine (14%) carried MRSP; in 55, we isolated MSSP (81%). Among the owners, in six cases (6%), dogs and humans had the same species (*S. pseudintermedius*). In other cases, the same species was not isolated from the dog and the owner.

Considering the number of animals carrying Methicillin-resistant *Staphylococcus pseudintermedius* compared to the sensitive ones, there was a statistical significance in relation to the possible animal-to-owner transmission in the MRSP.

Among the data collected from the animals, it is worth noting that all MRSP had previously used antimicrobials due to the chronicity of their dermatological condition. Additionally, it´s important to highlight that all animals sharing the same clone slept together in their owner’s beds, and in case of MRSP, their owners worked within a hospital environment.

**Fig. 1:**
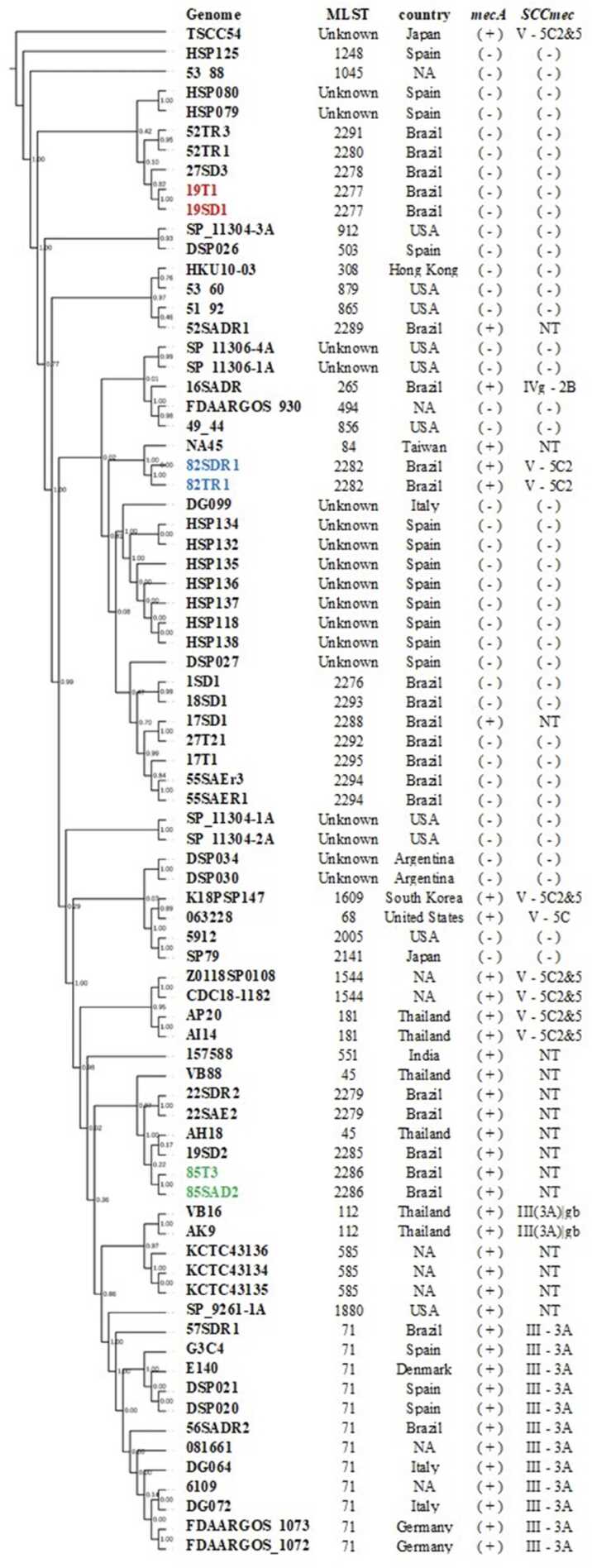
Phylogenetic three of the 23 isolates from this study and 55 genomes available in GenBank. MLST, Isolation country, in silico *mecA* detection, and SCC*mec* typing are shown in the table. * NT= Non-typable.

The phylogenetic tree showed that dogs and their owners had closely related isolates in three cases, two with MRSP samples (82SDR1 and 82T1 in blue; 85SAD2 and 85T3 in green) and one MSSP (19SD1 and 19T1in red) (Fig. 1). Also, in the two cases where MRSP was isolated from dogs and their owners, we identified the exact MRSP clone (MRSP ST2282-SCC*mec* V and MRSP ST2286 non-typable SCC*mec*). In the other case, the dog and its owner had the same clone of MSSP with the same sequence type (ST2277). The SNP analysis showed that the pairs dog/owner 82SDR1 and 82T1 shared 86 SNPs, 85SAD2 and 85T3 93 SNPs, and 19SD1 and 19T1 59 SNPs. Additionally, SNP analysis of other genomes grouped in the tree showed that isolates from different dogs 1SD1 and 18SD1 shared 7961 SNP, an isolate from a dog and a human 17SD1 and 27T21 9220 SNPs or even isolates from the same dog 55SAER1 and 55SAR3 152 SNPs or 22SAE2 and 22SDR2 205 SNPs (Table 1). This finding strongly suggests a possible transmission event occurred. Although both samples identified the same.

species in the other three cases, the samples were phylogenetically different.

Between the three possible transmitted pairs of samples and the other three pairs of different clones, we observed the presence of antimicrobial resistance genes: *blaZ* and *mecA* (B-lactams), *sdrM* (quinolones), *AAC(6′)-le-APH(2'')-la*, *APH*(3′)-IIIa, *aad*(6), *SAT*-4 (aminoglycosides), *sepA* (efflux pump), *dfrG* (trimethoprim), *ErmB* (Mlsb), *tet*(M) (tetracyclines), *CAT* (chloramphenicol) and *FosBx1* (fosfomycin) (Table 2). BRIG genome.

**Table 2:** Information regarding MLST, SCCmec type, and carriage of resistance genes of MRSP and MSSP strains isolated from infection sites among dogs and owners from Rio de Janeiro State (2018).

Sample	Origin	MLST	*SCCmec*	Resistance Profile	BLT	AGS	TET	TRI	CHL
17SD1	Canine-P	2288	NT	oxa,pen	*blaz, mecA*	*aph(3′)-iiia, aad(6), sat-4*	*tet(M)*	*ermB*	*dfrG*
17T1	Human-NF	2295	MSSP	pen, cip, mbf, eno	*blaz*				*dfrG*
19SD1	Canine-P	2277	MSSP						
19T1	Human-NF	2277	MSSP						
27SD3	Canine-P	2278	MSSP	pen					
27T2.1	Human-NF	2292	MSSP	pen	*blaz*		*tet(M)*		
52SADr1	Canine-O	2289	V	oxa, pen, clin, ery	*blaz, mecA*	*aph(3′)-iiia, aad(6), sat-4*	*tet(M)*	*ermB*	*dfrG*
52TR1	Human-NF	2280	MSSP	pen,clin,ery					
82SDr1	Canine-P	2282	V	oxa,pen	*blaz, mecA*	*aac(6′)-le-aph(2'')-la*			*dfrG*
82Tr1	Human-NF	2282	V	oxa,pen	*blaz, mecA*	*aac(6′)-le-aph(2'')-la*			*dfrG*
85SAD2	Canine-O	2286	NT	oxa,pen,cip,mbf,clin,ery,eno	*blaz, mecA*	*aac(6′)-le-aph(2'')-la,aph(3′)-iiia, aad(6)*		*ermB*	
85T3	Human-NF	2286	NT	oxa,pen,cip,mbf,clin,ery,eno	*blaz, mecA*	*aac(6′)-le-aph(2'')-la,aph(3′)-iiia, aad(6)*		*ermB*	

**Fig. 2:**
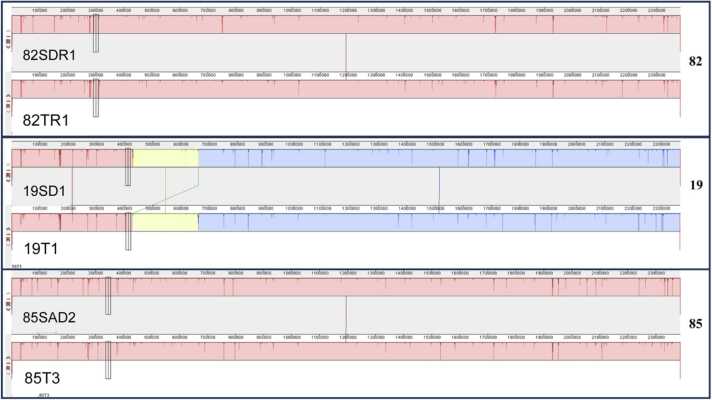
BRIG genome alignment: comparative analysis of whole genomes between canine samples and their owners.

**Fig. 3:**
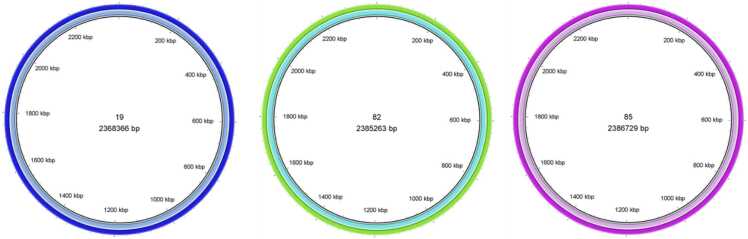
MAUVE genome alignment: comparative study of whole genomes between canine samples and their owners using ProgressiveMauve.

P = Pyoderma; O = Otitis; NF = Nasal Fossa; NT = non-typable; oxa = oxacillin; pen = penicillin; cip = ciprofloxacin; mbf = marbofloxacin; eno = enrofloxacin; clin = clindamycin; ery = erythromycin

alignment demonstrated that genomes from dogs and their owners were practily identical (Fig. 2), what could also be confirmed on MAUVE genome alignment (Fig. 3).

Considering the number of animals carrying Methicillin-resistant *Staphylococcus pseudintermedius* compared to the sensitive ones, there was a statistical significance (p < 0,005) in relation to the possible animal-to-owner transmission in the MRSP. The percentage of potential sharing of the same clone between owner and animal was higher in MRSPs (22%) compared to MSSPs (1,8%).

Among the data collected from the animals, it is worth noting that all dogs carrying MRSP had previously used antimicrobials due to the chronicity of their dermatological condition. Additionally, it´s important to highlight that all animals sharing the same clone slept together in their owner’s beds, and in case of MRSP, their owners worked within a hospital environment.

## Discussion

The literature characterizes *Staphylococcus pseudintermedius* as the predominant member of the canine normal microbiota, frequently encountered through isolation. The pathogenic propensity of *S. pseudintermedius* to cause infections in dogs has been firmly established [1], [2]. This study observed the persistence of the most prevalent species identified within infection samples, specifically those related to otitis and pyoderma. Furthermore, the extensive prevalence of colonization underscores the potential for transmission to humans [27], [28], [29], [30].

Research into human nasal colonization with *Staphylococcus pseudintermedius* within households where dogs are present has been documented in various regions, including Korea [31] and Portugal [32]. These studies revealed a human prevalence ranging from 3% to 4.5%, while canine prevalence ranged from 25% to 65.9%. Notably, a recent investigation by Cuny et al., 2022 reported a prevalence of 0.6% [33]. This prevalence closely mirrors the findings of our study, not only in human samples but also in samples from infected canines.

Furthermore, the prevalence of human infections caused by *S. pseudintermedius* could be higher than reported in the literature, mainly due to the striking similarities between *S. pseudintermedius* and *S. aureus*. Both species belong to the coagulase-positive Staphylococcus group and share specific biochemical characteristics. The potential for misidentification of *S. pseudintermedius* human infections as *Staphylococcus aureus* [16], [17] or *Staphylococcus intermedius* [18] could lead to underestimating the former's occurrence.

Staphylococci can persist on environmental surfaces for at least four months before inciting infections [34], [35]. Consequently, surfaces and items shared between dogs and their caretakers, such as beds or sofas, might act as carriers for transmitting staphylococci. While our study did not specifically assess this aspect, prior investigations have indeed focused on the contamination of surfaces, primarily within hospital settings, by *S. pseudintermedius* [36], [37].

After the phylogenetic analysis, detecting *S. pseudintermedius* presence in the dog-owner pairs involving six asymptomatic humans affirms the potential occurrence of zoonotic transmission in three instances. This is underscored by the shared sequence types (STs) and *SCCmec* elements and further reinforced by minimal single nucleotide polymorphisms (SNPs) observed. Evaluating antibiotic resistance profiles within the canine and human strains indicates a pervasive resistance encompassing antibiotics employed in clinical and veterinary therapeutic contexts. This broad dissemination of resistance genes among staphylococcal strains is a pivotal contributor to the emergence of extensively drug-resistant (XDR) and multidrug-resistant (MDR) bacteria, which can potentially impose significant threats upon human health.

Upon analyzing the MLST method, it becomes evident that most identified sequence types (STs) were previously undocumented globally. Despite originating from a confined geographical area, the isolates displayed remarkable diversity. While a recent study in Brazil spotlighted the prevalence of clone ST71 as the most widespread strain in Rio de Janeiro [11], our investigation did not reveal the sharing of this clone among dogs and their respective owners.

These findings highlight the significance of disseminating resistant strains between canines and their human counterparts. Given that colonization constitutes the initial phase in the progression toward infections, heightened attention should be directed toward the zoonotic implications of this species. Although infrequent, infections attributed to *S. pseudintermedius* have been reported in the scientific literature. Notable cases encompass endocarditis [38], bacteremia linked to medical implants [39], surgical site infections, cellulitis, and ulcers [12], including instances of diseases triggered by methicillin-resistant strains of *S. pseudintermedius* (MRSP) in humans. For example, a sinusitis case induced by clone ST71, the prevalent strain circulating in Europe, was documented [40]. Several years back, a review reported 24 human infections stemming from this species [41]. Notably, some of these cases implicated the family dog as a potential source of infection.

The current incidence of human infections caused by *S. pseudintermedius* remains largely obscure. Given its infrequent occurrence in human cases, the actual prevalence of colonization by this species remains undetermined [42]. Research indicates a notably higher colonization rate in dogs than other mammalian species. Unfortunately, investigations into human colonization are sparse and outdated. A study from 2009 revealed a human colonization rate of approximately 4.1% for *S. pseudintermedius*, a stark contrast to the roughly 30% colonization rate established for *S. aureus* [43]. Specific populations exhibit an elevated susceptibility to colonization by this species, particularly resistant strains. These populations encompass professionals such as veterinarians, groomers, and individuals caring for dogs afflicted by recurrent pyoderma [44].

It cannot be definitively stated which factors predispose the transmission of S. *pseudintermedius* between animals-owners and vice-versa. However, some points, such as close contact and owners who frequent or are involved in hospital environments, stand out among these points could favor this zoonotic transmission, especially of MRSPs [45]. These were also noted in this study.

## Conclusion

In conclusion, the current investigation substantiates the transmission of identical *S. pseudintermedius* clones between dogs and their respective owners. These findings are important in illuminating the zoonotic potential inherent to this species and the tangible risk of contracting illnesses linked to staphylococcal strains associated with animals. Moreover, the interchange of strains between dogs and their human counterparts raises the potential for transferring resistance and virulence genes between *S. pseudintermedius* and the more clinically significant *S. aureus*, a species of paramount importance in human medical contexts. Instances of this nature might be more prevalent than anticipated, particularly considering the intimate proximity shared between companion animals and their caregivers.

## Funding

This work was supported in part by grants from the Fundação Carlos Chagas Filho de Amparo à Pesquisa do Estado do Rio de Janeiro - 10.13039/501100004586FAPERJ (Projeto REDES: E26/211.554/2019; E-26/201.328/2021), Conselho Nacional de Desenvolvimento Científico e Tecnológico – 10.13039/501100003593CNPq (406057/2016-8; 443764/2018-2), Coordenação de Aperfeiçoamento de Pessoal de Nível Superior – 10.13039/501100002322CAPES and 10.13039/100000865Bill and Melinda Gates Foundation10.13039/100000865.

## Declaration of Competing Interest

The authors declare no conflict of interest.
